# Quality of Life and Psychological Distress among Patients with Small Renal Masses

**DOI:** 10.3390/jcm11143944

**Published:** 2022-07-07

**Authors:** Liliana Vartolomei, Andrei Cotruș, Camelia Stanciu, Cristian Delcea, Marco Tozzi, Elena Lievore, Felice Crocetto, Francesco Del Giudice, Giuseppe Lucarelli, Matteo Muto, Matteo Ferro

**Affiliations:** 1 I.O.S.U.D., George Emil Palade University of Medicine, Pharmacy, Sciences and Technology from Târgu Mureș, 540139 Târgu Mureș, Romania; liliana.vartolomei17@gmail.com; 2 Department of Urology, Medical University of Vienna, 1090 Wien, Austria; 3 Department of Psychology, Dimitrie Cantemir University, 540545 Târgu Mureș, Romania; andrei.cotrus@gmail.com (A.C.); cameliastanciu.psy@gmail.com (C.S.); 4 Department of Psychology, Tibiscus University, 300559 Timisoara, Romania; cristian.delcea.cj@gmail.com; 5 Department of Urology, European Institute of Oncology, IRCCS, 20141 Milan, Italy; marco.tozzi@ieo.it (M.T.); elena.lievore@ieo.it (E.L.); 6 Department of Maternal Infant and Urologic Sciences, ‘Sapienza’ University of Rome, 00161 Rome, Italy; francesco.delgiudice@uniroma1.it; 7 Department of Neurosciences, Human Reproduction and Odontostomatology, University of Naples Federico II, 80126 Naples, Italy; felice.crocetto@unina.it; 8 Andrology & Kidney Transplantation Unit, Department of Emergency & Organ Transplantation-Urology, University of Bari, 70121 Bari, Italy; giuseppe.lucarelli@inwind.it; 9 Department of Onco-Hematological Diseases, U.O.C. Radiotherapy-Azienda Ospedaliera San Giuseppe Moscati-(AV), 83100 Avellino, Italy; mattomuto@gmail.com

**Keywords:** nephrectomy, renal cancer, quality of life, small renal masses, active surveillance

## Abstract

Background: Quality of life (QoL) and psychological distress represent an important aspect of the daily life of cancer patients. The aim of this systematic review was to critically analyze available literature regarding QoL and psychological distress in patients with small renal masses (SRMs). (2) Methods: A systematic search of EMBASE, PUBMED and American Psychological Association (APA-net) was performed on 30 April 2022. Studies were considered eligible if they included patients with SRMs, had a prospective or retrospective design, included at least 10 patients, were published in the last 20 years, and assessed the QoL or psychological distress in patients that underwent active surveillance (AS) in comparison to those that underwent ablation/surgery treatments. (3) Results: The patients that underwent AS were statistically significantly older, with smaller renal masses than those that underwent surgery/ablation. A study showed a significant reduction in total scores of Short Form-12 (SF-12) among AS patients when compared to partial nephrectomy (PN) patients at enrollment (95.0 ± 15.8 vs. 99.1 ± 13.9), 2 years (91.0 ± 16.4 vs. 100.3 ± 14.3), and at 3 years (92.9 ± 15.9 vs. 100.3 ± 14.3), *p* < 0.05, respectively. That was mainly due to lower physical health scores. On the other hand, another study showed that AS patients with a biopsy-proven malignant tumor had a worse psychological distress sub-score (PDSS) compared to patients treated with surgery/ablation after biopsy. (4) Conclusions: It seems that there is an influence on QoL and psychological distress while on AS of SMRs. However, due to the low amount of available data, the impact of AS or active treatment on QoL or psychological distress of patients with small renal masses warrants further investigation.

## 1. Introduction

The incidence of renal cell carcinoma (RCC) is increasing, largely due to the widespread use of imaging. Most renal tumors are detected incidentally as small, asymptomatic masses (SRMs). Recommended therapeutic options for SRMs include active surveillance (AS), ablation, or surgery, mostly partial nephrectomy (PN) with either an open, laparoscopic or robotic approach [1].

AS is defined as the initial monitoring of tumor size by serial abdominal imaging (ultrasound (US), computer tomography (CT), or magnetic resonance imaging (MRI)) with delayed intervention reserved for tumors showing clinical progression during follow-up [2]. The concept of AS differs from the concept of watchful waiting (WW); which is indicated in the case of comorbidities that contraindicate an active treatment and do not require the strict follow-up used for AS.

A multicenter registry that included patients with SRMs and assessed the quality of life (QoL) of patients undergoing immediate treatment vs. AS showed that patients undergoing immediate intervention had higher QoL scores at baseline, specifically for physical health compared to those undertaking AS [3]. However, very little data exists on any long-term follow-up. In a cohort of 136 patients with biopsy-proven SRMs with renal cell carcinomas (RCCs) undergoing AS at a median follow-up of 5.8 years: 60 (44.1%) progressed, with 49 (82%) by rapid growth (volume doubling), seven (12%) increasing to >4 cm, and four (6.7%) by both criteria and only six (4.4%) patients developed metastases [4].

The aim of this systematic review was to critically analyze the available literature regarding QoL and psychological distress of patients with SRMs that underwent AS versus those that underwent surgery or ablation.

## 2. Materials and Methods

A systematic search of EMBASE, PUBMED and American Psychological Association APA-net data was performed on 30 April 2022, using combinations of the terms: quality of life (EXP) OR psychological distress (EXP) AND small renal masses (EXP) OR renal cancer (EXP). All original articles that fulfilled the inclusion criteria were included. Additional cross checking of reference lists to find additional studies was performed.

### 2.1. Protocol

The protocol of this systematic review followed the Cochrane handbook [5] and the Preferred Reporting Items for Systematic Reviews and Meta-analysis (PRISMA) criteria (https://www.prisma-statement.org (accessed on 1 March 2022)) [6].

### 2.2. Inclusion and Exclusion Criteria

Studies were considered eligible if they included patients with SRMs, had a prospective or retrospective design, included at least 10 patients, were published in the last 20 years, and assessed the QoL or psychological distress in patients that underwent AS in comparison to those that underwent ablation or surgery. The primary outcome was changes in QoL or psychological distress while on AS in comparison to ablation/surgery therapy. For each selected study, the following items were recorded: first author’s name, year of publication, country, design, age, number of patients, treatment option, inclusion criteria, questionnaires used, changes in QoL and follow-up. Two investigators independently conducted literature searches and extracted data from included full-text articles; disagreements were resolved by consensus.

## 3. Results

A total of 3 studies met the inclusion criteria, all coming from North America, two from the US [7,8] and one from Canada [9]. Studies that investigated QoL or psychological distress in patients with SRMs undergoing AS or WW or surgery or ablation without comparison between these approaches were excluded [10,11]. The included studies compared outcomes between patients that underwent AS and those that underwent surgery/ablation of SRMs (total no. patients 1654 with 810 (49%) on AS) (see Table 1). The patients that underwent AS were statistically significantly older and with smaller renal masses than those that underwent surgery/ablation. Goldberger et al. used Edmonton Symptom Assessment System-revised (ESAS-r) while Alam et al. [7] and Patel et al. [8] used Short Form 12 (SF-12) questionnaire to assess psychological distress and/or QoL. Alam [7] and Patel [8] cohorts were based on the Delayed Intervention and Surveillance for Small Renal Masses (DISSRM) Registry, which is a multi-institutional, prospective registry established in 2009 to evaluate the safety of AS compared to primary intervention for patients with SRMs, defined as clinical stage T1a tumors [12]. After a median 3-year follow-up, Alam et al. [7] showed a significant reduction in total SF-12 scores among AS patients when compared to PN patients at enrollment (95.0 ± 15.8 vs. 99.1 ± 13.9), 2 years (91.0 ± 16.4 vs. 100.3 ± 14.3), and 3 years (92.9 ± 15.9 vs. 100.3 ± 14.3), *p* < 0.05, respectively. That was mainly due to lower physical health scores (at 3 years 40.9 ± 12.5 vs. 47.1 ± 10.5), with no difference regarding mental component scores (at 3 years 52.0 ± 9.0 vs. 53.1 ± 7.6). On the other hand, Goldberger et al. [9] showed that while on AS, patients with a biopsy-proven malignant tumors had a worse psychological distress sub-score (PDSS) compared to patients treated with surgery/ablation after biopsy (11.4 vs. 6.1, *p* = 0.035), and at the last follow-up (13.2 vs. 5.9, *p* = 0.004).

## 4. Discussion

Psychological distress of patients with SRMs is of paramount importance when considering different treatment options. AS seems to burden oncological patients mainly in terms of physical well-being, and to a lesser extent in the psychological aspect. Generally, up to 77% of patients with renal cancer report anxiety and the need for supportive psychological care, irrespective of age, gender, tumor stage, presence of metastases, and type of treatment received [13,14]. Psychological distress is an important prognostic factor and was associated with worse survival and well-being [15].

When considering treatment options, literature shows that most SRMs are indolent malignances or benign lesions, and from a medical perspective, AS for SRMs has been proven equal to active treatment [12]. Due to these aspects, in patients of an older age or with reduced renal function and/or congenital or acquired solitary kidney, AS could be preferred, and clinicians tended to highlight this treatment option in these selected patients. This explains why in all studies patients undergoing AS are significantly older and have more comorbidities. However, as pointed out by Alam et al. [7] and Patel et al. [8], for this reason AS patients have worse QoL at baseline due to inferior health status, thus reflecting an unfavorable overall physical health with respect to patients treated with surgery or ablation. In fact, as Patel et al. points out, after diagnosis, AS patients did not have a worsening of QoL, and 40% of patients eligible for AS choose this management option. It is important to highlight that in this cohort, the AS program is standardized under the multi-institutional Delayed Intervention and Surveillance for Small Renal Masses (DISSRM) program, and a uniform consultation and programmed follow-up is of paramount importance for psychological well-being during AS.

Coping with cancer represents a daily challenge for patients; and in particular, dealing with renal cancer seems to correlate negatively with the health-related QoL [16,17]. This trend was also shown with other types of urological tumors. With prostate cancer (PCa), for example, it is reported that only a low percentage of diagnosed men choose AS, and preferences are usually dominated by perception of treatment efficacy and personal burden of that treatment [18]. Moreover, a general perception of fear of cancer may lead to an immediate need for active treatment when available [19]. Some findings, additionally, demonstrate that clinicians can create an involuntary barrier to AS programs due to personal preferences [20]. These data could also be applied to renal cancer, suggesting that patient counseling by physicians should always be exhaustive and encompass all treatment options with risks and benefits. As shown by Alam et al. [7], and Patel et al. [8], when patients are enrolled in a prospective and standardized program, mental health scores are similar between AS and active treatment groups.

On the other hand, with the advent of minimally invasive surgery, active surgical treatment is largely considered safe and effective. Minimally invasive nephron-sparing surgery leads to optimal oncologic outcomes with lower blood-loss and shorter hospital stays [21,22]. These data are coherent with the results regarding physical health and QoL after active treatment, as shown by Alam et al. [7].

Additionally, newer and larger data regarding ablative techniques are confirming the feasibility of this choice in SRMs [23,24] and due to the mini-invasiveness of the procedure, active treatment with this technique could be preferred, especially in patients with poor performance status, even if long-term follow up data are still lacking. Thanks to these advances, the burden of active treatment for renal cancer has been reduced and can consequently be perceived as low by patients, thus positively affecting psychological and physical well-being. For these reasons, clinician counseling is of paramount importance not only for decision making, but also for psychological acceptance of that decision.

Percutaneous biopsy may influence management of SRMs. As shown by Goldberg et al. [9], patients with biopsy proven malignant tumors had worse psychological distress during AS. Percutaneous biopsy has been showing a good accuracy in diagnosing malignant lesions and low percentage of side effects [25], and EAU Guidelines suggest performing it before AS [1] in order to select tumors at lower risk of progression based on grade and histotype. However, literature is controversial about psychological distress of patients with proven diagnosis of renal cancer. Data from the DISSRM registry [12] suggest that the psychological burden between patients undergoing AS versus active treatment does not differ. However, other retrospective studies showed that a non-negligible number of patients shift from AS to active treatment due to psychological distress of dealing with cancer [26,27]. Therefore, the choice of performing kidney biopsy should be weighted taking into consideration clinical options, patients’ preferences, and whether its results would impact management. Regarding cost of treatment, in a recent cost-analysis study it was shown that AS with possible delayed intervention, had the lowest total cost per patient in comparison to immediate PN, immediate radical nephrectomy or immediate ablation of SRMs [28].

Future prospects regarding treatment options for renal cancer are mainly evolving in the direction of digital imaging. Three-dimensional reconstruction seems to be useful for optimization of partial nephrectomies [29] and digital technology navigation during surgery can decrease operative time and complications [30] thus further reducing the burden of a surgical treatment. Advances in the field of percutaneous ablation could lead to an expansion of this technique especially in patients not suitable for surgical treatment who are not willing to undergo AS. On the other hand, despite all these advances, improved clarity and communication of treatment options should be of primary importance for the clinician, and especially for AS protocol and the appointment schedule needed should be clarified. These aspects could be of utmost importance in the decision making of patients with SRMs and their consequent psychological and physical well-being.

Limitations: The paucity of available studies does not allow drawing firm conclusions and suggests the need for further investigation regarding the psychological burden of patients with SRMs. Results from the included studies cannot be generalized for other populations from other cultures or geographical areas as QoL or psychological distress may differ by race [31]. The questionnaires used were different among studies and each has some limitations regarding specific items. For example, the ESAS-R assessed only symptom intensity and data about well-being and was not well-defined [32]. Since in all studies patients were not randomly assigned to surgical procedure vs. AS, factors that affected choice of surgical procedure that influenced QoL outcomes were not taken into consideration.

## 5. Conclusions

Patients with small renal masses that underwent active surveillance seem to have lower quality of life with respect to physical activity than their counterparts that underwent active treatment with some additional degree of psychological distress. However, available data comes only from two cohorts, and pertinent conclusions cannot be drawn. The impact of AS or active treatment on quality of life or psychological distress of patients with small renal masses warrants further investigation.

## Figures and Tables

**Table 1:** Studies that assessed the impact of quality of life (QoL) or psychological distress on patients with small renal masses (SRMs) that underwent active surveillance (AS) compared to ablation/surgical therapy.

No.	First Author	Year	Country	Study Design	No. Patients	Age Years	Treatment Option	Questionaires	Results	Follow-Up
1	Goldberg et al.	2020	Canada	single-center retrospective study	477 patients included between 2014 and 2017	56.0 (standard deviation [SD] 10.56) AS 51.9 (SD 11.16) Surgery/ablation	217 active surveillance (AS) and 260 surgery/ablation	Edmonton Symptom Assessment System-revised (ESAS-r)	AS-treated patients with a biopsy-proven malignant tumor had worse psychological distress sub-score (PDSS) compared to patients treated with surgery/ablation after biopsy (11.4 vs. 6.1, *p* = 0.035), and at last follow-up (13.2 vs. 5.9, *p* = 0.004).	surgery/ablation group 3.46 years vs. 2.03 years AS group
2	Alam et al.	2019	USA	Multi-left prospective registry	638 patients	61.3 [52.9–67.3] PN, 69.3 [55.3–75.5] RN, 71.8 [62.0–74.8] ablation, 70.6 [63.2–78.2] AS	231 (36.2%) partial nephrectomy (PN), 41 (6.4%) radical nephrectomy (RN), 27 (4.2%) ablation, and 339 (53.1%) AS.	Short Form 12 (SF12) QoL questionnaire at 6 and 12 months, and annually thereafter	QoL was lowest in AS patients due to lower physical health scores, but mental health scores were similar in all groups.	3 years IQR 1.6–5.0
3	Patel et al.	2016	USA	Multi-center prospective registry	539 patients included Between 1 January 2009 and 31 October 2015	62.0 (53.1–68.9) surgery/ablation 70.6 (62.6–78.6) AS	254 AS, 285 surgery/ablation, and 21 AS patients crossed over to delayed intervention.	Short Form 12 (SF12) QoL questionnaire at 6 and 12 months, and annually thereafter	Mental health, which includes domains of depression and anxiety was not adversely affected while on AS and improves over time after selecting a management strategy.	1.8 years IQR 0.3–3.0

## Data Availability

Not applicable.
